# Metabolic and Antioxidant Status, Telomere Lengths of Individuals With Prediabetes in Context of 3 Months of Insoluble Dietary Fibre Intake During the SARS‐CoV2 Pandemic

**DOI:** 10.1002/edm2.70210

**Published:** 2026-03-27

**Authors:** Mykola Khalangot, Dmytro Krasnienkov, Vitaliy Gurianov, Tamara Zakharchenko, Olexiy Barsukov, Kostiantyn Midlovets, Victor Kravchenko, Mykola Tronko

**Affiliations:** ^1^ Komisarenko Institute of Endocrinology and Metabolism Kyiv Ukraine; ^2^ Shupyk National Healthcare University of Ukraine Kyiv Ukraine; ^3^ Chebotariov Institute of Gerontology Kyiv Ukraine; ^4^ Bohomolets National Medical University Kyiv Ukraine; ^5^ Karazin Kharkiv National University Kharkiv Ukraine

**Keywords:** blood pressure, COVID‐19, glycated haemoglobin, impaired fasting glucose, insoluble fibre intervention, reduced glutathione

## Abstract

**Background:**

Insoluble fibre (IF) may reduce the risk factors of type 2 diabetes development, but evidence on its short‐term effects is limited. The influence of IF on oxidative stress markers, including reduced glutathione (GSH) and antioxidant enzymes, as well as on leukocyte telomere length (LTL), remains poorly studied. Coronavirus disease (COVID‐19) occurring before or during IF interventions may influence study outcomes.

**Methods:**

The study included 29 adults with impaired fasting glucose (IFG) who received 15 g/day of IF for 3 months. Seven participants had a recent history of COVID‐19, defined as PCR‐confirmed infection within 6 months before the intervention or COVID‐19‐like symptoms during the study. Fasting plasma glucose, 2‐h plasma glucose (2hPG) during a WHO oral glucose tolerance test, glycated haemoglobin (HbA1c) and GSH were assessed at baseline and after 3 months. Fat mass (FM), blood pressure (BP), LTL, superoxide dismutase and catalase (CAT) were also measured.

**Results:**

In participants without COVID‐19 (*n* = 22), IF intake was associated with reductions in 2hPG, HbA1c, FM and GSH, along with an increase in systolic BP. In the COVID‐19 group (*n* = 7), HbA1c decreased, while both systolic and diastolic BP increased. No significant changes in LTL, superoxide dismutase or CAT levels were observed in either group.

**Conclusion:**

Short‐term IF supplementation in individuals with IFG improved selected glycaemic parameters but was accompanied by unexpected reductions in GSH and increases in blood pressure. These findings indicate complex physiological responses to IF that warrant further investigation.

**Trial Registration:**

ClinicalTrials.gov identifier: NCT04283201

## Graphical abstract

This 3‐month study investigated the effects of 15 g/day insoluble fibre (IF) supplementation in 29 adults with impaired fasting glucose. While IF significantly reduced glycated haemoglobin (HbA1c) and 2‐h plasma glucose (2hPG), body fat in participants without recent COVID‐19, it also led to unexpected increases in blood pressure and a reduction in glutathione (GSH) levels.

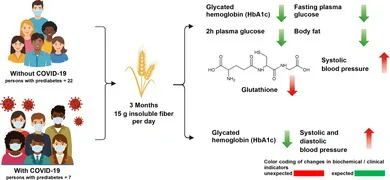

## 1 Introduction

Cereal fibre may have a positive effect on glycaemic metabolism, with the most pronounced effect observed in individuals with impaired fasting glucose (IFG) [1]. We recently demonstrated that 3 months of daily consumption of 15 g of insoluble fibre (IF) reduced fasting plasma glucose (FPG), 2‐h plasma glucose (2hPG) and glycated haemoglobin (HbA1c) in individuals with IFG defined by ADA criteria. A decrease in body mass index and fat mass (assessed by bioelectrical impedance analysis) was also reported. An unexpected finding was a small but statistically significant increase in blood pressure after the IF intervention [2].

There is evidence that dietary fibre (including IF) can reduce the production of reactive oxygen species after a meal. In 10 healthy adults, adding 28 g of IF to a proinflammatory high‐fat, high‐calorie (HFHC) meal prevented HFHC‐induced proinflammatory changes during the 5‐h follow‐up period [3]. Conversely, in another small study of six participants, dietary fibre supplementation decreased the antioxidative effect of a carotenoid and *α*‐tocopherol supplement in low‐density lipoproteins, likely due to reduced intestinal bioavailability of these antioxidants [4]. In both studies, IF exposure lasted only several hours. Therefore, the relationship between oxidative stress and dietary fibre intake remains controversial.

Given the metabolic and anthropometric effects of IF described above, it was reasonable to evaluate changes in oxidative stress markers after a longer IF intervention than those previously conducted by Ghanim H et al. [3] or Hoffmann J et al. [4]. We performed a 3‐month nutritional intervention during the SARS‐CoV‐2 pandemic, which could have influenced the outcomes—particularly weight loss—regardless of COVID‐19 severity [5]. Therefore, a history of coronavirus disease (COVID‐19) before or during the intervention may have affected our results.

The relevance of anthropometric measures and hyperglycaemia during COVID‐19 is supported by our earlier findings indicating that waist circumference is an independent risk factor for mortality [6]. A recent observational study also reported a positive association between leukocyte telomere length (LTL) and dietary fibre intake (Bountziouka V, et al.) [7]. In the present study, we were able to evaluate the response to the IF intervention according to COVID‐19 history. We present data on LTL and several oxidative stress markers, including reduced glutathione (GSH), superoxide dismutase (SOD) and catalase (CAT), which are appropriate targets based on prior IF studies [8] and recent COVID‐19 research [9]. GSH deficiency, resulting from oxidative stress caused by SARS‐CoV‐2 infection, has been documented in severe COVID‐19 cases and may be associated with increased mortality [10]. GSH is the major intracellular water‐soluble antioxidant and may be reduced both in diabetes and COVID‐19 [11].

We therefore tested whether an IF intervention in individuals with IFG would affect antioxidant levels, and how COVID‐19 history influenced the dynamics of glycaemic and body composition parameters during the intervention.

## 2 Methods

Study design: pilot, interventional, prospective, single‐factor, single‐group (before–after intervention). The study included individuals at increased risk of developing type 2 diabetes mellitus who consumed an insoluble dietary fibre supplement daily for 3 months. The study did not include a placebo group and therefore no randomisation was performed.

A detailed description of this intervention study can be found elsewhere [2]. In brief, the study describes 29 randomly selected rural residents with IFG as determined by ADA criteria [12]. Participants consumed 15 g of IF (VITACEL Wheat Fibre WF 600–30, containing 96% insoluble fibre) daily. Each participant received a monthly supply of 500 g of insoluble dietary fibre powder, distributed through a local primary healthcare worker. Participants were instructed to consume one level tablespoon three times daily with meals. Adherence to the recommended intake was monitored by issuing each new package only upon return of the previously distributed empty package. Subjects did not report taking any antioxidants or changing their antihypertensive treatment during the study after the 3‐month IF intervention.

The study intervention lasted 3 months. Evaluation of fasting plasma glucose (FPG) and 2hPG within the standard 75‐g oral glucose tolerance test (OGTT) recommended by the WHO, as well as HbA1c, reduced GSH, SOD, CAT, LTL and anthropometric indicators including body composition and blood pressure, was performed before and after the intervention. Examinations included waist and hip circumferences (WC, HC), height and weight, systolic and diastolic blood pressure (BP) measurements and fat mass (FM) assessed by bioelectrical impedance analysis. Plasma levels of total cholesterol (TC), high‐density lipoprotein cholesterol (HDL) and triglycerides (TG) were also evaluated.

### 2.1 Anthropometric Examinations

The baseline and 3‐monthly anthropometric examinations included measurements of weight to the nearest 0.1 kg (in light indoor clothes without shoes), height to the nearest 0.5 cm and waist and hip circumference. Fat mass was assessed by bioelectrical impedance analysis (BIA; Body Impedance Analyser Bodystat, Advantage Health Care Pty Ltd. Braeside Victoria 3195 Australia). Waist circumference (WC) was measured in a vertical position, with the patient's arms lowered along the body and relaxed, with a tight‐fitting centimetre tape at the level of the middle of the distance between the lower edge of the costal arch and the upper edge of the iliac crest, on exhalation. Hip circumference (HC) was measured in a vertical position, at the level of the most prominent point of the buttocks [13]. Body mass was measured using well‐tried electronic scales. WC and HC were measured with a flexible measuring tape at maximum transverse size in standing position. To measure arterial BP, we assessed the Korotkoff sounds using an operational BP monitor from the corresponding family medicine clinic. Blood pressure was measured twice, with an interval of 5 min. If there was a difference of more than 10 mm, we made a third measurement. The mean value of these 2/3 measurements was counted.

#### 2.1.1 Oxidative Stress Markers

Patients' blood (4 mL) was collected into vacutainers with anticoagulant K_2_EDTA. Blood was centrifuged with a density gradient of Histopaque‐1077 (Sigma, H8889‐100ML, USA) to obtain plasma and peripheral blood mononuclear cells (PBMCs). The samples were transferred to an ultra‐low temperature freezer at −80°C for long‐term (more than a day) storage.

Blood CAT (EC 1.11.1.6) activity was assessed in haemolysate prepared by subjecting whole blood to osmotic haemolysis with distilled water, followed by freezing and centrifugation. The haemolysate was incubated with a hydrogen peroxide solution, and CAT activity was determined spectrophotometrically by measuring the reaction product of residual hydrogen peroxide with ammonium molybdate. Calibration was performed using commercially available CAT (Sigma, C9322). Results were reported in units of enzyme activity per millilitre of blood [14].

The activity of SOD (EC 1.15.1.1) in the plasma was measured using an indirect spectrophotometric method based on the superoxide‐mediated oxidation of quercetin in an alkaline medium in the presence of tetramethylethylenediamine. This reaction causes a change in the colour of the solution, which is monitored at 406 nm. SOD activity, which inhibits quercetin oxidation, was quantified after a 20‐min incubation, with enzyme content determined from a calibration curve using commercially available SOD (Sigma, S9697). Results were expressed in units of SOD activity per litre [15].

Plasma GSH levels were determined using a spectrofluorometric method with orthophthalic aldehyde, which forms fluorescent products with GSH, excited at 350 nm and emitting at 420 nm. GSH concentration was calculated using a calibration curve from a commercial GSH assay kit (Sinbias, Ukraine) and reported in micromoles per litre [16].

The GSH as well as CAT or SOD measurements were carried out using a Thermo electron corporation Varioscan Flash spectrofluorometer (USA, Waltham).

#### 2.1.2 Leukocyte Telomere Length Measurement

LTL was measured using quantitative real‐time polymerase chain reaction (qPCR) according to the method by Cawthon [17]. DNA was extracted from peripheral blood mononuclear cells (PBMCs) using the phenol‐chloroform method. The qPCR reaction mix was prepared with a commercial reagent kit (Solis BioDyne) and included betaine (Sigma, USA) at a final concentration of 1 M. Telomere primers (telg and telc) were used at a final concentration of 450 nM each, and albumin primers (albu and albd) at 250 nM each, in a master mix. Thermal cycling included: 12 min at 95°C; two cycles of 15 s at 94°C, 15 s at 49°C; 32 cycles of 15 s at 94°C, 10 s at 62°C, 15 s at 74°C with signal acquisition, 10s at 84°C and 15 s at 88°C with signal acquisition. Calibration curves were generated using four concentrations of reference DNA, prepared by serial dilution, in duplicates covering a 27‐fold range. DNA samples were analysed in triplicates, and amplification curves were generated using an Opticon Monitor 3 Real‐Time PCR System (Bio‐Rad, Hercules, CA, USA). LTL was expressed as the T/S ratio, the ratio of telomere repeats copy number (T) to single‐copy gene (scg) copy number (S). PCR was performed using a Bio‐Rad Chromo4 thermal cycler (USA, Hercules). Amplification curves were generated using the Opticon Monitor 3 software.

PCR was performed using a Bio‐Rad Chromo4 thermal cycler (USA, Hercules). Amplification curves were generated using the Opticon Monitor 3 software.

List of primers used for MMqPCR:

- telg: 5′–ACACTAAGGTTTGGGTTTGGGTTTGGGTTTGGGTTAGTGT–3′;
- telc: 5′–TGTTAGGTATCCCTATCCCTATCCCTATCCCTATCCCTAACA–3′;
- albd: 5′–GCCCGGCCCGCCGCGCCCGTCCCGCCGGAAAAGCATGGTCGCCTGTT– 3′;
- albu: 5′–CGGCGGCGGGCGGCGCGGGCTGGGCGGAAATGCTGCACAGAATCCTTG–3′.

#### 2.1.3 Biochemical Measurements

Blood samples were collected on an empty stomach (after an overnight fast of at least 8 h) and 2 h after glucose solution consumption (75 g of glucose was dissolved in 200 mL of water). Blood plasma collected on EDTA, without traces of haemolysis, was separated from blood cells no later than 30 min after blood sampling by centrifugation.

Levels of FPG, plasma glucose 2‐h after 75 g of glucose load (2hPG), plasma TG, plasma TC, plasma high‐density lipoprotein cholesterol (HDL‐C) were measured using colorimetric tests and a Microlab‐300 spectrophotometer (‘Vital Scientific’, Netherlands) with a wavelength of 500–550 nm. Glucose levels were determined by the glucose oxidase endpoint method using the ‘Liquick Cor‐Glucose’ kit, TG by the glycerophosphorus oxidase endpoint method using the ‘Liquick Cor‐TG’ kit, TC by the esterase and cholesterol oxidase using the ‘Liquick Cor‐CHOL’ kit, HDL‐C by the precipitation method with phosphotungstic acid using the ‘Cormay HDL’ and ‘Liquick Cor‐CHOL’ kits. All biochemical kits used were manufactured by ‘Cormay’, Poland. HbA1c was assessed using a rapid analyser and Clover A1c test cartridges (‘Infopia Co., Ltd’, Republic of Korea) using the boronate affinity method for HbA1c fixation.

#### 2.1.4 COVID‐19 History

Individuals with a PCR‐confirmed diagnosis within 6 months before the start of the intervention (*n* = 5) or symptoms of COVID‐19 (*n* = 2) during the intervention were assigned to the group with a history of COVID‐19 (*n* = 7). So, we recorded seven cases of COVID‐19 (with mild symptoms that did not require hospitalisation). During the IF intervention, IF consumption was not stopped or changed. Twenty‐two people were in the group without COVID‐19 history.

#### 2.1.5 Bioethical Aspects

The study protocol No. 32‐KE dated 02/27/2020 was approved by the ethics committee of the Institute of Endocrinology and Metabolism, National Academy of Medical Sciences, Ukraine. Written informed consent was obtained from each participant before the study began. All of those involved, including family physicians, took part in the study as volunteers.

Statistical analysis was performed using the MedCalc Statistical Software, version 23.2.6 (MedCalc Software Ltd., Ostend, Belgium; https://www.medcalc.org; 2025). Quantitative variables were presented as the mean (M) ± standard deviation (SD) for normally distributed data or as the median (Me) with interquartile range (Q1–Q3) for non‐normally distributed data.

The Mann–Whitney *U* test was used to compare two independent samples. Changes in parameters after the intervention were assessed using the paired samples *t*‐test or the non‐parametric Wilcoxon signed‐rank test, as appropriate. Spearman's rank correlation analysis was also performed. To control the false discovery rate in multiple hypothesis testing, the Benjamini–Hochberg procedure was applied [18]. Statistical significance was set at *p* < 0.05.

## 3 Results

While glycaemic (FPG, 2hPG, HbA1c), fat mass (BIA fat) and reduced glutathione (GSH) indicators decreased statistically significantly, systolic and diastolic blood pressure levels increased after 3 months of IF intervention. Other investigated anthropometric and biochemical indicators, as well as SOD, CAT and LTL, did not change. FPG reduction was not confirmed according to the Benjamini–Hochberg procedure for multiple hypothesis testing (Table 1).

**TABLE 1:** Metabolic and antioxidant markers, blood pressure and leukocyte telomere length (LTL) levels of 29 individuals with IFG before and after 3 months of insoluble dietary fibre (IF) intervention.

Variables	Before IF intervention Mean **±** SD/Median (Q1–Q3)	After IF intervention Mean **±** SD/Median (Q1–Q3)	*p*	p*
FPG, mmol/L	5.65 ± 0.54	5.33 ± 0.69	**0.042**	0.080
2hPG, mmol/L	5.76 ± 1.37	5.05 ± 1.33	**0.005**	**0.014**
HbA1c, %	6.2 ± 0.31	5.92 ± 0.37	**< 0.001**	**0.004**
WC, cm	99.1 ± 13.65	96.48 ± 11.37	0.063	0.107
HC, cm	111.03 ± 10.04	110.72 ± 11.24	0.843	0.843
BIA fat, %	42.5 (33.2–48.3)	41.5 (31.025–48.250)	**0.005**	**0.014**
BIA fat, kg	36.85 (27–44)	36.2 (23.450–43.025)	**0.001**	**0.004**
BIA TBW, %	44.2 (40–50.4)	44.9 (39.650–53.150)	**0.006**	**0.015**
ТС, mmol/L	5.68 (5.080–6.320)	5.7 (5.020–6.307)	0.581	0.706
HDL‐C, mmol/L	1.41 ± 0.27	1.4 ± 0.24	0.708	0.752
TG, mmol/L	1.035 (0.720–1.340)	0.99 (0.778–1.258)	0.160	0.227
Systolic BP, mmHg	125.27 ± 25.72	134.41 ± 24.88	**0.001**	**0.004**
Diastolic BP, mmHg	77.43 ± 10.24	82.41 ± 13.34	**0.008**	**0.017**
GSH, μM/L	8.87 ± 0.75	8.14 ± 0.88	**0.001**	**0.004**
SOD, U/L	7.3 ± 0.82	6.95 ± 0.77	0.132	0.204
CAT, U/mL	922 (834–963.250)	953 (867.250–998)	0.175	0.229
LTL, T/S ratio	0.174 (0.133–0.243)	0.181 (0.121–0.204)	0.642	0.728

*Note:* *p*‐(paired samples *t*‐test or non‐parametric Wilcoxon *T*‐test) is presented in the context of the IF intervention, p*‐with correction according to the Benjamini–Hochberg procedure for multiple hypothesis testing.

The age of individuals in the group without a history of COVID‐19 was higher than in the group with a history of COVID‐19 during the IF intervention. 2hPG was lower after the IF intervention only in the non‐COVID‐19 group, but HbA1c levels were lower after the IF intervention in both groups. A decrease in body fat (BIA) and GSH levels was found only in the group without a history of COVID‐19 during the IF intervention. An increase in systolic BP after the IF intervention was recorded in both groups, and diastolic BP increased only in the group with a history of COVID‐19.

With correction for multiple hypothesis testing we found a decrease in HbA1c after IF intervention in both groups, divided according to COVID‐19 history, while an increase in Systolic BP and decrease in 2hPG, BIA fat and GSH was confirmed only in the group without a history of COVID‐19 (Table 2).

**TABLE 2:** Metabolic and antioxidant markers, leukocyte telomere lengths (LTL) levels of individuals with IFG before and after 3 months of insoluble dietary fibre (IF) intervention depending on COVID‐19 history.

Variables	No COVID‐19, *n* = 22				COVID‐19, *n* = 7			
	Before IF intervention Mean **±** SD/Median (Q1–Q3)	After IF intervention Mean **±** SD/Median (Q1–Q3)	*p*	p*	Before IF intervention Mean **±** SD/Median (Q1–Q3)	After IF intervention Mean **±** SD/Median (Q1–Q3)	*p*	p*
Age, years	63 (56.25–67.0)				47 (43.25–58.25)			
FPG, mmol/L	5.7 ± 0.49	5.37 ± 0.62	0.060	0.110	5.49 ± 0.7	5.23 ± 0.93	0.450	0.750
2hPG, mmol/L	5.66 ± 1.45	4.86 ± 1.22	**0.007**	**0.023**	6.09 ± 1.07	5.64 ± 1.56	0.418	0.749
HbA1c, %	6.25 ± 0.3	6.03 ± 0.33	**0.003**	**0.017**	6.03 ± 0.28	5.56 ± 0.22	**0.002**	**0.034**
WC, cm	100.0 ± 13.5	96.6 ± 10.8	0.054	0.113	96 ± 14.8	96 ± 13.9	> 0.999	> 0.999
HC, cm	110.6 ± 10.3	110.5 ± 11.7	0.943	0.958	112.43 ± 9.61	111.29 ± 10.61	0.529	0.749
BIA fat, %	42.5 (29.7–48.275)	41.2 (29.3–47.5)	**0.008**	**0.023**	46.6 (34.92–49.27)	46.2 (33.47–48.72)	0.469	0.749
BIA fat, kg	36.3 (21.075–43.1)	34.25 (18.2–42.6)	**0.001**	**0.017**	42 (31.82–44.15)	39.3 (30.07–43.07)	0.297	0.749
BIA TBW, %	44.2 (40.5–55.325)	45.2 (42.4–59)	**0.010**	**0.024**	40 (39.4–48.775)	42.3 (38.87–49.95)	0.345	0.749
ТС, mmol/L	5.82 (5.152–6.3)	6.02 (5.230–6.460)	0.775	0.941	5.54 (4.530–6.192)	5.67 (4.945–5.895)	0.813	0.864
HDL‐C, mmol/L	1.41 ± 0.21	1.43 ± 0.22	0.958	0.958	1.42 ± 0.45	1.33 ± 0.32	0.618	0.750
TG, mmol/L	1.01 (0.745–1.240)	0.98 (0.78–1.24)	0.188	0.246	1.13 (0.620–1.565)	1.17 (0.680–1.525)	0.578	0.750
Systolic BP, mmHg	125.6 ± 24.9	135.6 ± 24.8	**0.004**	**0.017**	124.29 ± 30.34	130.71 ± 26.84	**0.049**	0.278
Diastolic BP, mmHg	77.74 ± 9.16	80.91 ± 12.11	0.102	0.173	76.43 ± 14.06	87.14 ± 16.8	**0.019**	0.162
GSH, μM/L	8.83 ± 0.78	8.04 ± 0.92	**0.003**	**0.017**	9.01 ± 0.71	8.49 ± 0.69	0.160	0.680
SOD, U/L	7.34 ± 0.86	6.96 ± 0.73	0.187	0.246	7.18 ± 0.7	6.94 ± 0.95	0.488	0.749
CAT, U/mL	915 (830–970)	942 (874–997)	0.149	0.230	922 (869–961)	953 (856–993)	0.800	0.864
LTL, T/S ratio	0.167 (0.124–0.237)	0.18 (0.107–0.207)	0.949	0.958	0.174 (0.155–0.253)	0.181 (0.145–0.185)	0.469	0.749

*Note:* *p*‐(paired samples *t*‐test or non‐parametric Wilcoxon *T*‐test) is presented in the context of the IF intervention. p*‐with correction according to the Benjamini–Hochberg procedure for multiple hypothesis testing.

The results of the comparison of these parameters between the age groups (age ≤ 60 years, *n* = 14 and age > 60 years, *n* = 15) are presented in Table 3. After the IF intervention, HbA1c, absolute fat mass assessed by BIA and GSH levels decreased in both age groups. In the Age ≤ 60 years group, blood pressure did not increase significantly, although a trend towards higher systolic blood pressure was observed (*p* = 0.061). In the > 60‐year age group, glucose tolerance, as assessed by 2hPG levels, improved. Relative fat mass and TG levels decreased, whereas relative total body water and both systolic and diastolic blood pressure increased.

**TABLE 3:** Metabolic and antioxidant markers, telomere lengths of individuals with IFG before and after 3 months of insoluble dietary fibre (IF) intervention depending on age.

Variables	Age ≤ 60 years, *n* = 14				Age > 60 years, *n* = 15			
	Before IF intervention Mean **±** SD/Median (Q1–Q3)	After IF intervention Mean **±** SD/Median (Q1–Q3)	*p*	p*	Before IF intervention Mean **±** SD/Median (Q1–Q3)	After IF intervention Mean **±** SD/Median (Q1–Q3)	*p*	p*
FPG, mmol/L	5.58 ± 0.61	5.31 ± 0.67	0.230	0.360	5.72 ± 0.45	5.35 ± 0.74	0.120	0.190
2hPG, mmol/L	5.95 (4.690–6.787)	4.845 (4.04–5.95)	0.173	0.306	6.25 (5.050–7.090)	5.135 (3.750–5.540)	**0.022**	0.075
HbA1c, %	6.2 (5.925–6.375)	5.75 (5.5–5.9)	**0.003**	0.051	6.2 (6–6.5)	6.1 (5.725–6.3)	**0.042**	0.079
WC, cm	96 ± 13.77	93.5 ± 11.53	0.603	0.683	102.2 ± 13.26	99.27 ± 10.86	0.073	0.124
HC, cm	111.47 ± 9.66	110.29 ± 10.78	0.421	0.551	110.6 ± 10.72	111.13 ± 12.02	0.755	0.864
BIA fat, %	42.3 (35.92–47.92)	41.55 (36.4–48.4)	0.064	0.218	43.1 (29.67–49.72)	40.6 (29.87–47.3)	**0.041**	0.079
BIA fat, kg	37.4 (27.95–43.85)	34.2 (25.2–43)	**0.048**	0.218	36.3 (22.77–45.35)	36.2 (21.55–43.32)	**0.005**	**0.034**
BIA TBW, %	43.9 (39.85–48.42)	43.55 (39.4–50)	0.136	0.306	44.2 (40.5–56.32)	47.1 (42.62–57.17)	**0.038**	0.079
ТС, mmol/L	5.82 (5.062–6.327)	5.82 (5.020–6.280)	0.391	0.551	5.54 (5.152–6.3)	5.58 (5.062–6.362)	> 0.999	> 0.999
HDL‐C, mmol/L	1.45 ± 0.32	1.41 ± 0.26	0.540	0.656	1.38 ± 0.22	1.4 ± 0.23	0.680	0.864
TG, mmol/L	1.04 (0.730–1.153)	1.14 (0.790–1.250)	0.683	0.683	1.01 (0.767–1.588)	0.97 (0.773–1.220)	**0.027**	0.077
Systolic BP, mmHg	110 (93.75–132.5)	115 (105–150)	0.061	0.218	125 (112.5–145)	135 (130–158.7)	**0.008**	**0.034**
Diastolic BP, mmHg	75 (66.25–83.75)	77.5 (65–90)	0.180	0.306	80 (76.250–85)	85 (80–90)	**0.004**	**0.034**
GSH, μM/L	8.98 ± 0.88	8.23 ± 0.99	**0.044**	0.218	8.77 ± 0.62	8.06 ± 0.79	**0.008**	**0.034**
SOD, U/L	7.44 ± 0.88	6.97 ± 0.7	0.145	0.306	7.18 ± 0.76	6.93 ± 0.85	0.488	0.691
CAT, U/mL	873.5 (816.5–961)	961.5 (860–1009.5)	0.126	0.306	953 (870–972)	926 (867–972.5)	0.937	0.996
LTL, T/S ratio	0.157 (0.139–0.237)	0.175 (0.137–0.186)	0.670	0.683	0.177 (0.123–0.302)	0.182 (0.112–0.261)	0.762	0.864

*Note:* *p*‐(paired samples *t*‐test or non‐parametric Wilcoxon *T*‐test) is presented in the context of the IF intervention. p*‐with correction according to the Benjamini–Hochberg procedure for multiple hypothesis testing.

After correction according to the Benjamini–Hochberg procedure for multiple hypothesis testing, IF‐related changes could be confirmed for fat mass, systolic and diastolic blood pressure and GSH levels in the group aged > 60 years (Table 3). In both almost quantitatively equal age groups, IF intervention is associated with a decrease in chronic glycaemia (HbA1c) and GSH levels, while an increase in blood pressure is statistically proven only in the older group.

In addition to this analysis in age groups, we conducted a Spearman correlation analysis on the possible relationship between the dynamics (∆) of these variables and age as a continuous (1 year) variable (Table 4). A positive association with age was found for ΔHbA1c, negative for Δ2hPG and ΔTG. Changes in glutathione levels (ΔGSH) did not show a correlation with age (Table 4), which is consistent with the analysis in age groups (Table 3).

**TABLE 4:** Correlation assessment of some anthropometric, metabolic, antioxidant markers and telomere lengths Dynamics (∆Var = Var_after_‐Var_before_) related to IF intervention of 29 individuals with prediabetes.

Variables	Age, year	∆FPG	∆2hPG	∆HbA1	∆TG	∆WC	∆SBP	∆DBP	∆Fat	∆TBW	∆GSH	∆SOD	∆LTL
Age, year			**−0.387**	**0.405**	**−0.422**								
∆FPG		—	—		—	—	—	—	**0.393**	—	—	—	—
∆2hPG	**−0.387**	—	—		—	—	—	—	—	—	**−0.464**	—	—
∆HbA1	**0.405**												
∆TG	**−0.422**	—	—		—	—	—	—	—	—	—	—	**−0.389**
∆WC		—	—		—	—	—	—	**0.454**	**−0.409**	—	—	—
∆SBP		—	—		—	—	—	**0.63**	—	—	—	—	—
∆DBP		—	—		—	—	**0.63**	—	—	—	—	—	—
∆Fat		**0.393**	—		—	**0.454**	—	—	—	**−0.903**	—	—	—
∆TBW		—	—		—	**−0.409**	—	—	**−0.903**	—	—	—	—
∆GSH		—	**−0.464**		—	—	—	—	—	—	—	**0.514**	—
∆SOD		—	—		—	—	—	—	—	—	**0.514**	—	—
∆ LTL		—	—		**−0.389**	—	—	—	—	—	—	—	—

*Note:* Spearman's rank correlation coefficient.

**FIGURE 1:**
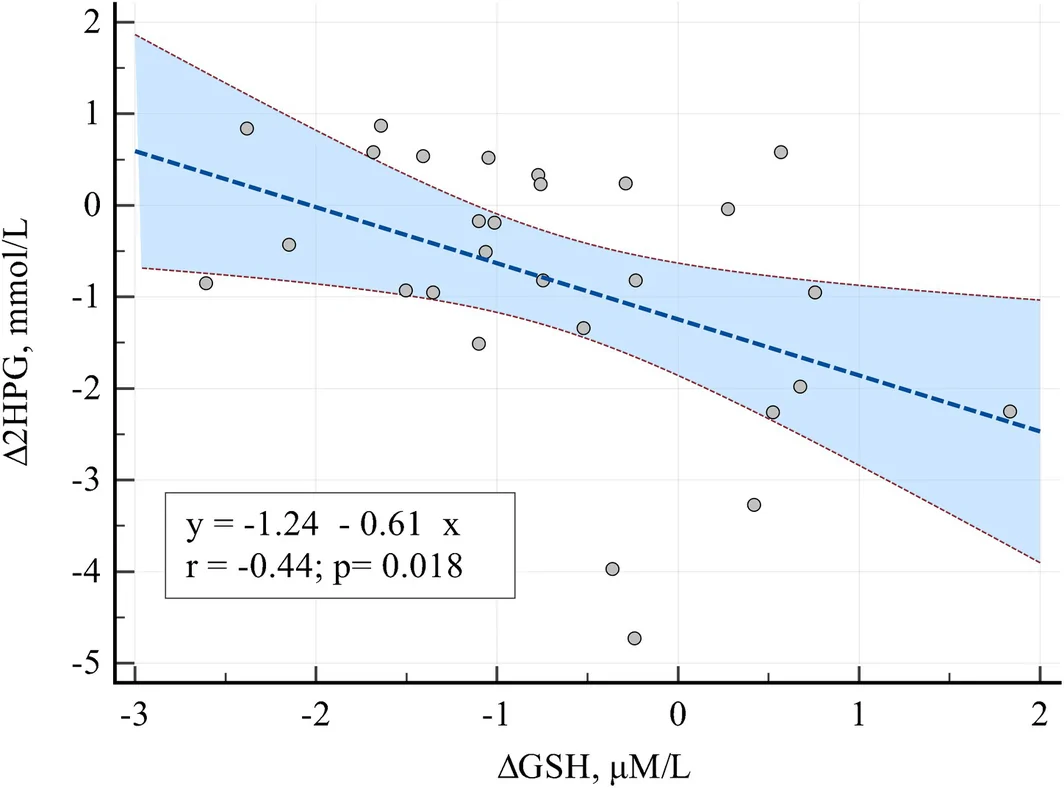
Negative relationship of ΔGSH with the dynamics of Δ2hPG: regression line (with 95% CI).

**FIGURE 2:**
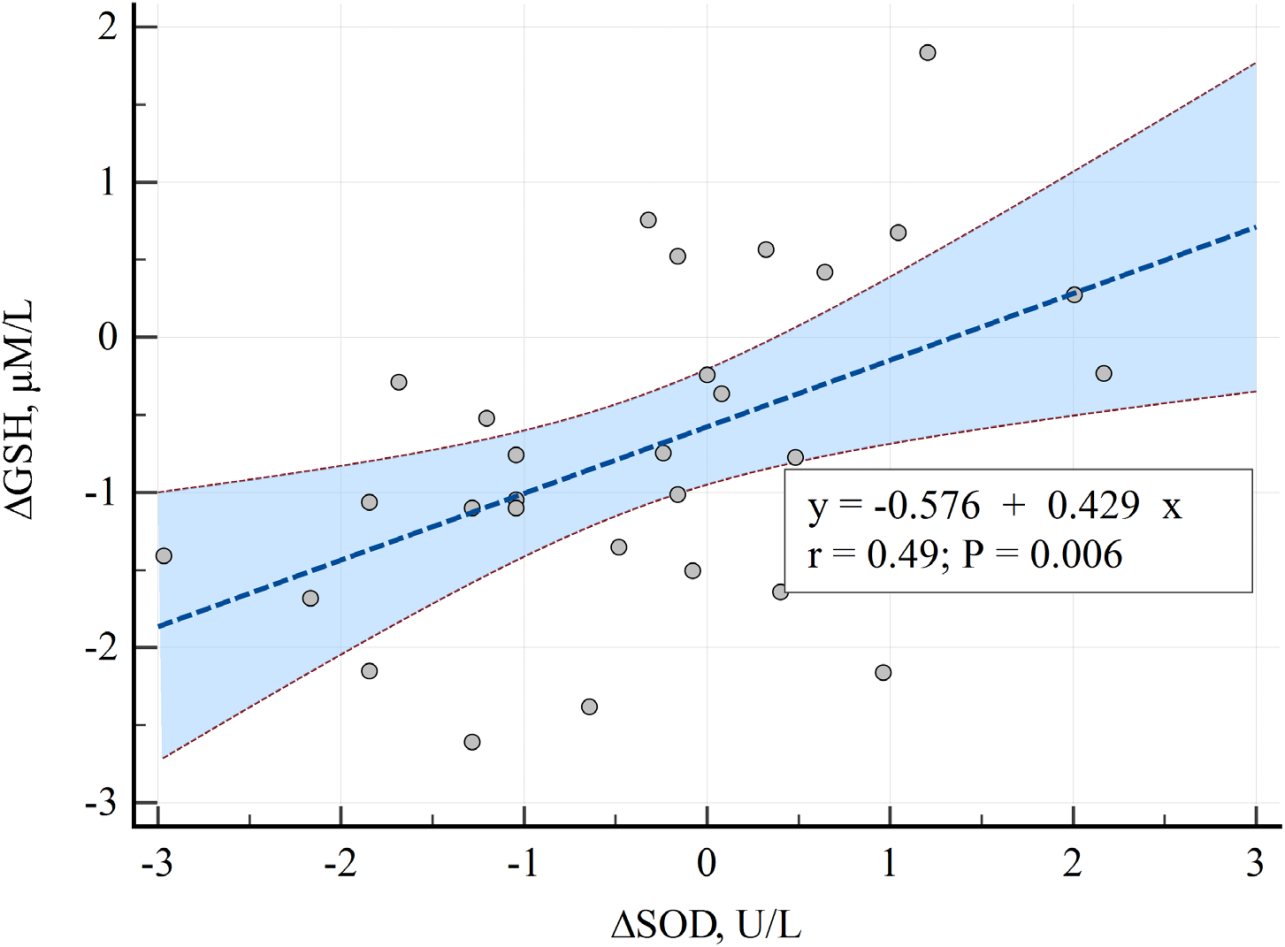
Positive relationship of ΔGSH with changes in ΔSOD: regression line (with 95% CI).

Correlation assessment of the intervention dynamics of ΔGSH revealed its negative relationship with the dynamics of Δ2hPG and a positive correlation with changes in ΔSOD: Spearman's rank correlation coefficients −0.464 and 0.514, respectively (Table 4). Figure 1 and Figure 2 show the corresponding regression lines (with 95% CI).

The ‘raw’ data are presented in a basic table containing all individual data and codes, Table S1.

## 4 Discussion

In the non‐COVID‐19 group, IF intervention was associated with a decrease in 2hPG, HbA1c, FM and GSH levels, and an increase in systolic blood pressure. In the COVID‐19 group, HbA1c levels decreased after IF, while systolic and diastolic blood pressure increased. LTL, superoxide dismutase and CAT levels did not show significant changes in association with COVID‐19 or IF intervention.

We tried to take into account the age factor (Age ≤ 60 vs. > 60 years) when analysing the effect of IF intervention on the studied parameters of metabolism and antioxidant defence. It is noteworthy that in both almost equal age groups, IF intervention is associated with a decrease in chronic glycaemia (HbA1c) and GSH levels, while an increase in blood pressure is statistically proven only in the older group.

COVID‐19 history may have influenced some of the effects of the IF intervention, but the lack of the expected effect may be explained by the small size of this group. On the other hand, the lack of the expected effect of an IF intervention may be a real consequence of COVID‐19 history (e.g., changes in appetite or physical activity levels).

It is important for us to note that we confirmed the previously observed metabolic effects of the IF intervention and found a decrease in GSH in the group without a history of COVID‐19.

Thus, after excluding the possible influence of COVID‐19, we confirmed a decrease in glycaemic indices and body fat after a 3‐month IF intervention. Unexpected results of such an intervention were also confirmed or revealed, namely an increase in BP and a decrease in GSH levels.

Malaysian researchers studied the effect of 3‐month use of IF (15 g daily) on symptoms and laboratory indicators in 27 patients with rheumatoid arthritis (RA). In addition to the reduction of RA symptoms, significant reductions in circulating inflammatory biomarkers, improvement of oxidative stress indices and antioxidant status (except GSH) were observed in the IF group [8]. In contrast to Latif et al., we found no changes in SOD and CAT activity (increase and decrease, respectively) after 12 weeks of IF consumption. However, we found a positive correlation (*p* < 0.006) between changes in GSH and SOD during the IF intervention (Figure 2).

That is, the decrease in GSH that we found, which is an antioxidant whose deficiency is associated with COVID‐19 and diabetes [10, 11], is correlated with a decrease in the activity of SOD, an enzyme known for its antioxidant properties. In this study, we found an improvement in glucose tolerance (a decrease in 2hPG) after a 12‐week IF intervention, but this decrease was negatively associated (*p* = 0.018) with a decrease in GSH: the greater the decrease in GSH, the less the decrease in 2hPG (Figure 1). Therefore, in the context of our results, the decrease in GSH can hardly be considered a positive consequence of IF intervention. Los et al. suggest that dietary supplementation with the glutathione precursors glycine and N‐acetyl‐L‐cysteine may be a practical approach to managing the oxidative stress associated with both diabetes and COVID‐19 [11]. Thus, we can hypothesise that this dietary supplement may prevent the decrease in GSH levels observed after IF intervention.

At the same time, it is known that the relationship between the pro‐oxidant and antioxidant properties of GSH and SOD depends on the nature of the compound and specific conditions [19]. There is at least one other study [20] that found a decrease in GSH after consuming the flavonoid quercetin in elderly patients with metabolic syndrome. Currently, we can only speculate about the mechanism of IF‐related GSH depletion. Since the use of IF can disrupt the absorption of some elements [21], one possible reason for the decrease in GSH may be the decrease in the trace element selenium (Se) due to the IF intervention. Se is a cofactor of glutathione peroxidase, which reduces hydrogen peroxide (H_2_O_2_) to H_2_O using GSH as a co‐substrate. There is a direct relationship between Se and GSH in the blood of adults who received and did not receive Se [22], so testing the selenium hypothesis may be appropriate.

After a 12‐week IF intervention, we found an increase in BP and a decrease in GSH levels. GSH is involved in nitric oxide metabolism and BP control (dilates blood vessels). In 2000, it was reported that oxidative stress and arterial hypertension were produced in rats by oral administration of buthionine sulfoximine (BSO), which induces GSH depletion, indicating that oxidative stress may induce hypertension. The BSO‐treated group showed a 3‐fold decrease in tissue GSH content, a marked elevation in blood pressure and a significant reduction in the urinary excretion of the NO metabolite nitrate plus nitrite, which suggests depressed NO availability [23]. In humans, GSH concentration in erythrocytes was significantly decreased in hypertensive patients when compared with normotensive subjects [24], while in the whole blood of persons receiving pharmacological treatment of arterial hypertension, the GSH concentration increased [25].

## 5 Conclusion

Evidence for improvements in glycaemic control and body composition following the IF intervention in individuals with IFG was confirmed in participants without a history of COVID‐19, whereas HbA1c decreased independently of COVID‐19 status. Changes in blood pressure and GSH levels could not be attributed to prior COVID‐19 infection. Notably, an increase in blood pressure associated with the IF intervention was statistically significant in participants aged 60 years and older.

The absence of a control group and adequate randomization limits the ability to infer a causal relationship between the IF intervention and the observed changes in the studied outcomes. Therefore, the present findings should be interpreted as exploratory. Nevertheless, the concomitant decrease in GSH and increase in blood pressure observed in association with the IF intervention may be discussed within existing pathophysiological frameworks and warrants further investigation.

## Author Contributions

M.K.: conceptualization, writing – review and editing; D.K.: investigation, writing – review and editing; V.G.: software, formal analysis, methodology; T.Z.: investigation, writing – review and editing; O.B.: investigation; K.M.: visualisation, writing – review and editing; V.K.: formal analysis, data curation; M.T.: conceptualization, supervision.

## Funding

This study was supported by the National Research Foundation of Ukraine.

Grant# 2021.01/0213 ‘Study of the course and consequences of COVID‐19 in patients with diabetes mellitus and the impact of SARS‐CoV‐2 infection on the rate of biological aging’.

## Ethics Statement

The study protocol No. 32‐KE dated 02/27/2020 was approved by the ethics committee of the Institute of Endocrinology and Metabolism, National Academy of Medical Sciences, Ukraine. Written informed consent was obtained from each participant before the study began.

## Conflicts of Interest

The authors declare no conflicts of interest.

## Supporting information


**Table S1:** Basic table containing all individual data and codes. All letters ‘F’ in the column headings represent data at the time of recording after the 3‐month Insoluble Fibre intervention. The abbreviations are no different from those used in the main text. The units of the quantitative data correspond to the description in the main text. Qualitative data (COVID‐19, current smoking and blood pressure treatment) were encoded as binary variables: 0 for absence and 1 for presence.

## Data Availability

The data that support the findings of this study are available on request from the corresponding author. The data are not publicly available due to privacy or ethical restrictions.
